# Analysis of Substrate Specificity and Kinetics of Cyclic Nucleotide Phosphodiesterases with N’-Methylanthraniloyl-Substituted Purine and Pyrimidine 3′,5′-Cyclic Nucleotides by Fluorescence Spectrometry

**DOI:** 10.1371/journal.pone.0054158

**Published:** 2013-01-14

**Authors:** Daniel Reinecke, Frank Schwede, Hans-Gottfried Genieser, Roland Seifert

**Affiliations:** 1 Institute of Pharmacology, Hannover Medical School, Hannover, Germany; 2 Biolog Life Science Institute, Bremen, Germany; German Research School for Simulation Science, Germany

## Abstract

As second messengers, the cyclic purine nucleotides adenosine 3′,5′-cyclic monophosphate (cAMP) and guanosine 3′,5′-cyclic monophosphate (cGMP) play an essential role in intracellular signaling. Recent data suggest that the cyclic pyrimidine nucleotides cytidine 3′,5′-cyclic monophosphate (cCMP) and uridine 3′,5′-cyclic monophosphate (cUMP) also act as second messengers. Hydrolysis by phosphodiesterases (PDEs) is the most important degradation mechanism for cAMP and cGMP. Elimination of cUMP and cCMP is not completely understood, though. We have shown that human PDEs hydrolyze not only cAMP and cGMP but also cyclic pyrimidine nucleotides, indicating that these enzymes may be important for termination of cCMP- and cUMP effects as well. However, these findings were acquired using a rather expensive HPLC/mass spectrometry assay, the technical requirements of which are available only to few laboratories. N’-Methylanthraniloyl-(MANT-)labeled nucleotides are endogenously fluorescent and suitable tools to study diverse protein/nucleotide interactions. In the present study, we report the synthesis of new MANT-substituted cyclic purine- and pyrimidine nucleotides that are appropriate to analyze substrate specificity and kinetics of PDEs with more moderate technical requirements. MANT-labeled nucleoside 3′,5′-cyclic monophosphates (MANT-cNMPs) are shown to be substrates of various human PDEs and to undergo a significant change in fluorescence upon cleavage, thus allowing direct, quantitative and continuous determination of hydrolysis *via* fluorescence detection. As substrates of several PDEs, MANT-cNMPs show similar kinetics to native nucleotides, with some exceptions. Finally, they are shown to be also appropriate tools for PDE inhibitor studies.

## Introduction

The cyclic purine nucleotides cAMP and cGMP are established second messengers known to regulate numerous cellular functions [1], [2], [3], [4]. The role of cyclic pyrimidine nucleotides has been discussed controversially in terms of natural occurrence [5], [6], [7], [8], generation [5], [6], degradation [9], [10], [11] and function [12], [13], [14]. Recent studies have shown that the cyclic pyrimidine nucleotides cUMP and cCMP are present in mammalian cells at levels similar to cAMP and cGMP [15], that they are generated by soluble guanylyl cyclase in presence of Mn^2+^ [16], [17] and that their production is regulated by nitric oxide and bicarbonate [16], [18]. Various effector proteins for cCMP and cUMP have been identified [17], [19], [20], [21], [22]. cCMP mediates vasodilatation and inhibits platelet aggregation *via* cGMP kinase I [23]. Furthermore, bacterial “adenylyl” Cyclase toxins act as cytidylyl- and uridylyl cyclases [24]. Taken together, cCMP and cUMP possess several properties that are characteristic for second messengers.

However, the assumed second messenger role of cCMP and cUMP requires the existence of effective mechanisms of elimination for cCMP and cUMP. Referring to cAMP and cGMP, PDEs limit their signals in terms of time and space [25], [26], [27]. Concerning pyrimidine nucleotides, two cCMP-degrading PDEs were postulated [9], [10]. A cUMP-cleaving PDE was found by Hardman and Sutherland in homogenates of bovine and canine hearts [28], but the molecular identities of cCMP- and cUMP-degrading PDEs have remained elusive. Recently, we have shown that several enzymes belonging to the eleven classes of well characterized human PDEs [25] are capable of hydrolyzing not only cAMP or cGMP but also cUMP, whereas PDE1B, PDE2A, PDE3A, PDE4B, PDE5A, PDE8A and PDE9A were not able to cleave cCMP [29]. These findings were obtained using a highly sensitive and specific but extensive HPLC-MS method that is available only to few laboratories.

The use of fluorescence-labeled substrates is very common in the examination of enzymatic reactions. MANT as fluorescent probe is a suitable tool to label various nucleotides for enzymatic studies, as it is rather small and attached to the nucleotide’s ribose, rendering steric inhibition of enzymatic reactions more unlikely than modifications at the base or the phosphoryl moiety [30], [31]. However, MANT-substituted nucleoside 5′-triphosphates (MANT-NTPs) act as inhibitors of mammalian and bacterial nucleotidyl cyclases [32], [33], [34], [35]. In contrast, *Escherichia coli* GTPase Era hydrolyzes MANT-GTP [36]. MANT-substituted nucleotides have been used to analyze various nucleotide/protein interactions e.g. with wheatgerm cap binding proteins [30], catalytic subunits of membranous adenylyl cyclase [37] and eukaryotic release factor 3 [38]. MANT has been attached to the bacterial second messenger cyclic di-guanosine monophosphate (c-di-GMP), being a substrate for a PDE from *Mycobacterium smegmatis* [39]. The more lipophilic di-MANT-c-diGMP is meanwhile commercially available. Hiratsuka used MANT- and ANT-substituted cAMP and cGMP as substrates of bovine heart PDE [40], and Johnson *et al.* detected activity of rabbit brain PDE on 2′-O-(N’-methylanthraniloyl)-cGMP (MANT-cGMP) [41], but there is a lack of comprehensive data on the interaction of purine and particularly pyrimidine MANT-cNMPs with human PDEs, and ideal fluorescence detection conditions for MANT-cNMPs are controversial in the literature.

In the present study, we describe the synthesis of various new MANT-substituted purine and pyrimidine 3′,5′-cyclic nucleotides. We show that MANT-cNMPs are substrates of different human PDEs - with 2′-O-(N’-methylanthraniloyl)-cCMP (MANT-cCMP) being a remarkable exception - and that their turnover can be quantified by direct fluorescence detection, thus rendering them appropriate tools to study the substrate specificity of PDEs.

## Materials and methods

### Materials

**Figure 1:**
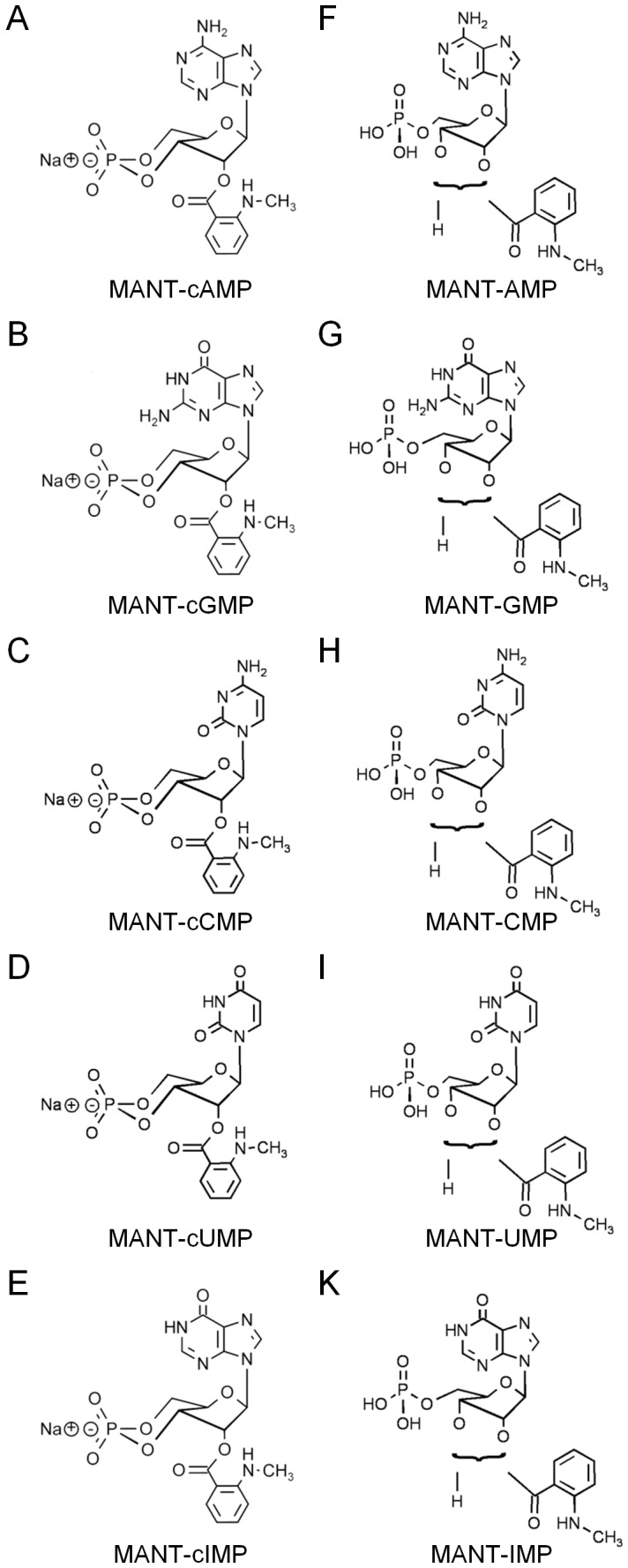
MANT-substituted nucleotides used for the fluorescence assay. **A-E** show the sodium salts of the MANT-cNMPs. **A**: MANT-cAMP, **B**: MANT-cGMP, **C**: MANT-cCMP, **D**: MANT-cUMP, **E**: MANT-cIMP. **F-K** display the MANT-NMPs. In MANT-NMPs, the MANT-group spontaneously isomerizes between the 2′- and 3′-O-ribosyl position. **F**: MANT-AMP, **G**: MANT-GMP, **H**: MANT-CMP, **I**: MANT-UMP, **K**: MANT-IMP.

Purified recombinant human PDEs 1B (purity >50%), 3A (purity >50%), 5A (purity >70%) and 9A (purity >80%) expressed in *Spodoptera frugiperda* Sf9 cells were obtained from BPS Bioscience (San Diego, CA). MANT-cGMP, 2′-O-(N’-Methylanthraniloyl)-cAMP (MANT-cAMP), MANT-cCMP, 2′/3′-O-(N’-Methylanthraniloyl)-GMP (MANT-GMP) (purity >99% each, determined by analytical HPLC at λ_max_), cCMP, inosine 3′,5′-cyclic monophosphate (cIMP), cUMP, cytidine 5′-monophosphate (CMP), inosine 5′-monophosphate (IMP) and uridine 5′-monophosphate (UMP) were provided as sodium salts by Biolog Life Science Institute (Bremen, Germany). 2′/3′-O-(N’-Methylanthraniloyl)-AMP (MANT-AMP) was obtained from Jena Bioscience (Jena, Germany). Structures of the MANT-cNMPs and 2′/3′-O-(N’-methylanthraniloyl)-NMPs (MANT-NMPs) that were used in this study are shown in figure 1. N-methylisatoic anhydride and cAMP were purchased from Sigma (Taufkirchen, Germany). Calmodulin was purified from calf brain as described [42]. All other reagents were of analytical grade or the best grade available from commercial suppliers.

### Synthesis of MANT-nucleotides: General Procedures

Analytical HPLC was performed with a LaChrom Elite system with EZ ChromElite software version 3.3.1 SP1 and consisted of a L-2200 autosampler, a L-2130 pump, a L-2455 UV/Vis diode array detector, a L-2350 column oven, and a D 7500 chromato-integrator (all VWR/Hitachi, Hannover, Germany). The stationary phase was ODS-A 12 nm, S-11 µm (YMC, Dinslaken, Germany) in a 250×4.6 mm stainless steel column. Preparative MPLC was accomplished with a C-605 pump (Büchi, Essen, Germany), a preparative K 2001 UV-detector (Knauer, Berlin, Germany) and a L200E analog recorder (Linseis, Selb, Germany). Merck LiChroprep RP-18 6 nm, 15–25 µm (Merck-Hitachi) in a 410×50 mm glass column (Kronlab, Dinslaken, Germany) was used for isolation, desalting and preparation the sodium salts of the nucleotides. Mass spectra were recorded with an Esquire LC 6000 spectrometer (Bruker Daltonics, Bremen, Germany) in the ESI-MS mode with 50% propanol-2/49.9% water/0.1% formic acid (v/v/v) as matrix. UV-spectra were recorded with a Helios β spectrometer (Spectronic Unicam, Leeds, UK) in aqueous phosphate buffer, pH 8. Nucleotides were quantified and aliquoted using the extinction coefficient of the N’-methylanthraniloyl group in MANT-guanosine and MANT-GTP at 355 nm [43].

### General Protocol of MANT-NMP Synthesis

3,500 µmol nucleoside 5′-monophosphate (NMP) was dissolved in 34 mL H_2_O and 17 mL acetonitrile in a 100 mL two-necked flask equipped with pH-electrode and reflux condenser and heated to 35°C. 8.750 µmol (2.5 eq.). N-methylisatoic anhydride was added in one portion and the resulting brown suspension was stirred and maintained at pH 9 by addition of 2.5 N NaOH. The progress of reaction was monitored by analytical HPLC (11% acetonitrile, 20 mM triethyl ammonium formate (TEAF) buffer, pH 6.9, 1.5 mL/min). After completion of the reaction (1–2 h) the raw mixture was extracted with CHCl_3_ (3 x 100 mL), concentrated under reduced pressure to remove residual acetonitrile and filtered through a 5 cm membrane of regenerated cellulose, 0.45 µm pore size (Sartorius, Göttingen, Germany). The resulting filtrate was purified by MPLC. After pre-equilibration with 100 mM NaH_2_PO_4_, pH 6.5 and desalting with 100% water, the particular MANT-NMP, sodium salt, was eluted as mixture of diastereomers with a gradient from 0% to 10% (v/v) isopropanol. Product-containing fractions with purities >99% (HPLC) were pooled, evaporated under reduced pressure to produce the MANT-NMP.

#### 2′/3′-O-(N’-Methylanthraniloyl)-CMP (MANT-CMP)

Yield: 355 µmol, 10.1%; purity: 99.7% (HPLC). UV/Vis pH 8 (λ_max_/ε): 254 nm/15,400; 355 nm/5,700. Empirical formula: C_17_H_19_N_4_O_9_P·Na_2_ (MW: 500.31), C_17_H_21_N_4_O_9_P (MW: 456.36, free acid). ESI/MS (isopropanol/water) pos. mode: m/z = 501 [M+2Na-H]^+^, 100%; m/z = 523 [M+3Na-2H]^+^, 25%; neg. mode: m/z = 455 [M-H]^-^, 100%; m/z = 477 [M+Na-2H]^-^, 10%.

#### 2′/3′-O-(N’-Methylanthraniloyl)-UMP (MANT-UMP)

Yield: 2,141 µmol, 61.2%; purity: 99.9% (HPLC). UV/Vis pH 8 (λ_max_/ε): 256 nm/17,800; 355 nm/5,700. Empirical formula: C_17_H_18_N_3_O_10_P·Na_2_ (MW: 501.30), C_17_H_20_N_3_O_10_P (MW: 457.33, free acid). ESI/MS (isopropanol/water) pos. mode: m/z = 502 [M+2Na-H]^+^, 100%; neg. mode: m/z = 456 [M-H]^-^, 100%; m/z = 478 [M+Na-2H]^-^, 10%.

#### 2′/3′-O-(N’-Methylanthraniloyl)-IMP (MANT-IMP)

Yield: 1,941 µmol, 55.5%; purity: 99.0% (HPLC). UV/Vis pH 8 (λ_max_/ε): 250 nm/20,800; 355 nm/5,700. Empirical formula: C_18_H_18_N_5_O_9_P·Na_2_ (MW: 525.32), C_18_H_20_N_5_O_9_P (MW: 481.36, free acid). ESI/MS (isopropanol/water) pos. mode: m/z = 526 [M+2Na-H]^+^, 100%; m/z = 548 [M+3Na-2H]^+^, 25%; neg. mode: m/z = 480 [M-H]^-^, 100%; m/z = 502 [M+Na-2H]^-^, 10%.

#### Synthesis of 2′-O-(N’-Methylanthraniloyl)-cUMP (MANT-cUMP)

555 µmol cUMP, sodium salt and 16,500 µmol (∼30 eq.) N-methylisatoic anhydride were suspended in 10 mL H_2_O in a 100 mL two-necked flask and processed according to the general protocol for MANT-NMP analogs as outlined above. Analytical HPLC for reaction control (15% isopropanol, 20 mM TEAF-buffer, pH 6.9, 1.0 mL/min, UV 262 nm). Yield: 449 µmol, 80.9%; purity: 99.5% (HPLC). UV/Vis pH 8 (λ_max_/ε): 255 nm/18,900; 355 nm/5,700. Empirical formula: C_17_H_17_N_3_O_9_P·Na (MW: 461.30), C_17_H_18_N_3_O_9_P (MW: 439.32, free acid). ESI/MS (isopropanol/water) pos. mode: m/z = 462 [M+2Na-H]^+^, 100%; m/z = 440 [M+H]^+^, 90%;m/z = 484 [M+3Na-2H]^+^, 60%; neg. mode: m/z = 438 [M-H]^-^, 100%.

#### Synthesis of 2′-O-(N’-Methylanthraniloyl)-cIMP (MANT-cIMP)

1,400 µmol cIMP, sodium salt and 21,000 µmol (∼15 eq.) N-methylisatoic anhydride were suspended in 15 mL H_2_O and 7.5 mL acetonitrile in a 100 mL two-necked flask and processed according to the general protocol for MANT-NMP analogs. Analytical HPLC for reaction control (24% acetonitrile, 20 mM TEAF-buffer, pH 6.9, 1.0 mL/min, UV 250 nm). Yield: 808 µmol, 57.7%; purity: 99.6% (HPLC). UV/Vis pH 8 (λ_max_/ε): 250 nm/20,500; 355 nm/5,700. Empirical formula: C_18_H_17_N_5_O_8_P·Na (MW: 485.33), C_18_H_18_N_5_O_8_P (MW: 463.34, free acid). ESI/MS (isopropanol/water) pos. mode: m/z = 508 [M+2Na-H]^+^, 100%; neg. mode: m/z = 462 [M-H]^-^, 100%.

### End Point Assay for PDE Activity Analysis *via* Direct Fluorescence Detection

Using human PDEs 3A, 5A and 9A, the experiments were carried out in a reaction buffer containing final concentrations of 50 mM Tris/HCl pH 7.5, 8.3 mM MgCl_2_ and 1.7 mM EDTA. Assaying PDE 1B, further ingredients to the buffer were 100 nM calmodulin and 100 µM CaCl_2_, constituting concentrations known to provide a sufficient activation of PDE1 [29], [44], [45]. To buffer the concentration of free calcium ions, 100 µM EGTA was added to the buffer as described in [45] Concentration of free Ca^2+^ ions was calculated as 2.9 µM using Ca-Mg-ATP-EGTA calculator v1.0 (http://www.stanford.edu/~cpatton/CaMgATPEGTA-NIST.htm) which is about 30-fold the basal calcium concentration in most cell types [46]. The EC_50_ for activation by calcium varies from 0.27 to 3.02 µM for various PDE1 isoforms [25]. Each MANT-cNMP was added to the buffer at a final concentration of 10 µM unless otherwise noted. The reaction was started by adding a PDE to each sample at final concentrations of 0.5 µg/ml (PDE1B), 0.7 µg/ml (PDE3A), 0.4 µg/ml (PDE5A) or 1 µg/ml (PDE9A). Each combination of available MANT-cNMP and PDE was studied. A 10 µM solution of each MANT-cNMP and MANT-NMP in reaction buffer was used as control, being substrate and product standard for the subsequent fluorescence analysis and calculations. Final sample volume was 200 µl. Incubation was performed at a temperature of 25°C for various reaction times between 10 to 60 minutes. The reaction was terminated by heating the samples to 58°C for 3 minutes, as heating at higher temperatures caused a depression of MANT-cNMP fluorescence in the consecutive fluorescence detection (data not shown). Precipitates were sedimented *via* centrifugation at 4°C and 20,000 g for 10 minutes. 100 µl of the supernatant were transferred to an UV-permeable synthetic quartz glass 96 well microtiter plate type 730.009B-QG by Hellma (Müllheim, Germany). 200 µl of dimethyl sulfoxide (DMSO) were added to these samples in some cases to improve fluorescence intensity. Fluorescence detection was performed using a Synergy 4 fluorimeter from BioTek (Bad Friedrichshall, Germany). Excitation wavelengths from 280 nm up to 350 nm were used to detect emission spectra of substrate standard, product standard and samples containing the respective PDE preparation in triplicates.

### Continuous Fluorescence Detection Assay

Samples containing reaction buffer and MANT-substituted cyclic nucleotides at a final concentration of 10 µM (unless otherwise noted) were transferred to an UV-permeable synthetic quartz glass 96 well microtiter plate along with the substrate standards of 10 µM MANT-cNMP and the product standards of 10 µM MANT-NMP. To start the reaction process, the respective PDE preparations was added to the samples. As in the end point assay, each combination of MANT-cNMP and PDE was examined. Final sample volume was 100 µl. Fluorescence measurements were started immediately after initiation of the reaction with excitation at 280 nm and emission detection at 450 nm and repeated every 20 to 30 seconds during the reaction process, plotting the fluorescence intensity against time. K_m_ and V_max_ values were calculated exemplary from the resulting graphs.

### Thin Layer Chromatography

50 µl of samples gained with the end point analysis method described above were applied to a silica gel glass plate for thin layer chromatography (TLC), type 60, item no. 105721, obtained from Merck (Darmstadt, Germany). For this assay, only DMSO-free samples were used. A solution containing 60% (v/v) of isopropanol, 30% (v/v) of a 25% (m/v) ammonium hydroxide solution and 10% (v/v) of water were used as mobile phase. Chromatography was carried out for 105 minutes. Plates were dried and spots were visualized using a 366 nm UV-lamp. The spots were marked and their respective retardation factors (R_f_-values) were determined. Each spot was scraped off the plate and eluted with 200 µl Tris/HCl 0.5 mol/l pH 8.0. Insoluble particles were sedimented *via* centrifugation at 4°C and 20,000 g for 10 minutes. To 100 µl of the supernatant 200 µl of DMSO were added, followed by fluorescence spectrum detection with excitation at 280 nm.

## Results

### Evaluating Fluorescence Detection Conditions

The fluorescence properties of each MANT-cNMP were tested in comparison to the corresponding MANT-NMP. Excitation spectra were recorded from 260 to 410 nm detecting emission at 440 nm (figure S1). Whereas every MANT-substituted nucleotide showed a fluorescence maximum at 440 nm when excited with 360 to 370 nm, the difference in fluorescence of each couple of MANT-cNMP and MANT-NMP was rather small in this range. MANT-cIMP/MANT-IMP turned out to be an exception. However, MANT-cGMP and to lesser extent MANT-CMP showed a shoulder in the curve progression when excited at 280 to 290 nm, where their fluorescence was clearly distinct from their corresponding nucleotides. MANT-cIMP and MANT-IMP exhibited a very low fluorescence in this range.

Adding increasing DMSO concentrations to the samples amplified the fluorescence of each tested MANT nucleotide substantially and led to a shift of maximum emission to shorter wavelengths. This is shown for MANT-cAMP exemplary in figure S2. Although adding DMSO did not alter the curve progression of the excitation spectra fundamentally for any MANT-substituted nucleotide (figure S1), it increased the discriminatory power between cNMP- and NMP-analogs in some cases. This was especially true for MANT-cAMP/MANT-AMP and MANT-cUMP/MANT-UMP. Detection of emission spectra was then been performed (figure S3) using various excitation wavelengths that were promising based on the analysis of the respective excitation spectra shown in figure S1 to investigate detection parameters providing ideal discriminatory power (calculated as ratio of MANT-cAMP/MANT-AMP fluorescence intensity). In conclusion, these studies led to the emission detection parameters displayed in table 1 providing the best discriminatory power for each MANT-cNMP/MANT-NMP couple respectively.

**Table 1:** Fluorescence detection parameters providing the best discriminatory power for each couple of MANT nucleotides.

	Excitation Wavelength	Emission Wavelength	DMSO	Discriminatory Power
MANT-cAMP/MANT-AMP	280 nm	440–450 nm	yes	1.39±0.09
MANT-cGMP/MANT-GMP	280 nm	440–450 nm	no	1.93±0.28
MANT-cCMP/MANT-CMP	290 nm	440–450 nm	no	0.61±0.04
MANT-cUMP/MANT-UMP	290 nm	440–450 nm	yes	0.62±0.14
MANT-cIMP/MANT-IMP	350 nm	440–450 nm	no	0.81±0.06

Discriminatory power is given as ratio of fluorescence intensity of each MANT-cNMP divided by the fluorescence intensity of the respective MANT-NMP when applying the detection parameters stated in the table. An emission wavelength of 440 nm was used for calculation.

### End Point Assay for PDE Activity Analysis *via* Direct Fluorescence Detection and Calculation of Turnover Rates

**Figure 2:**
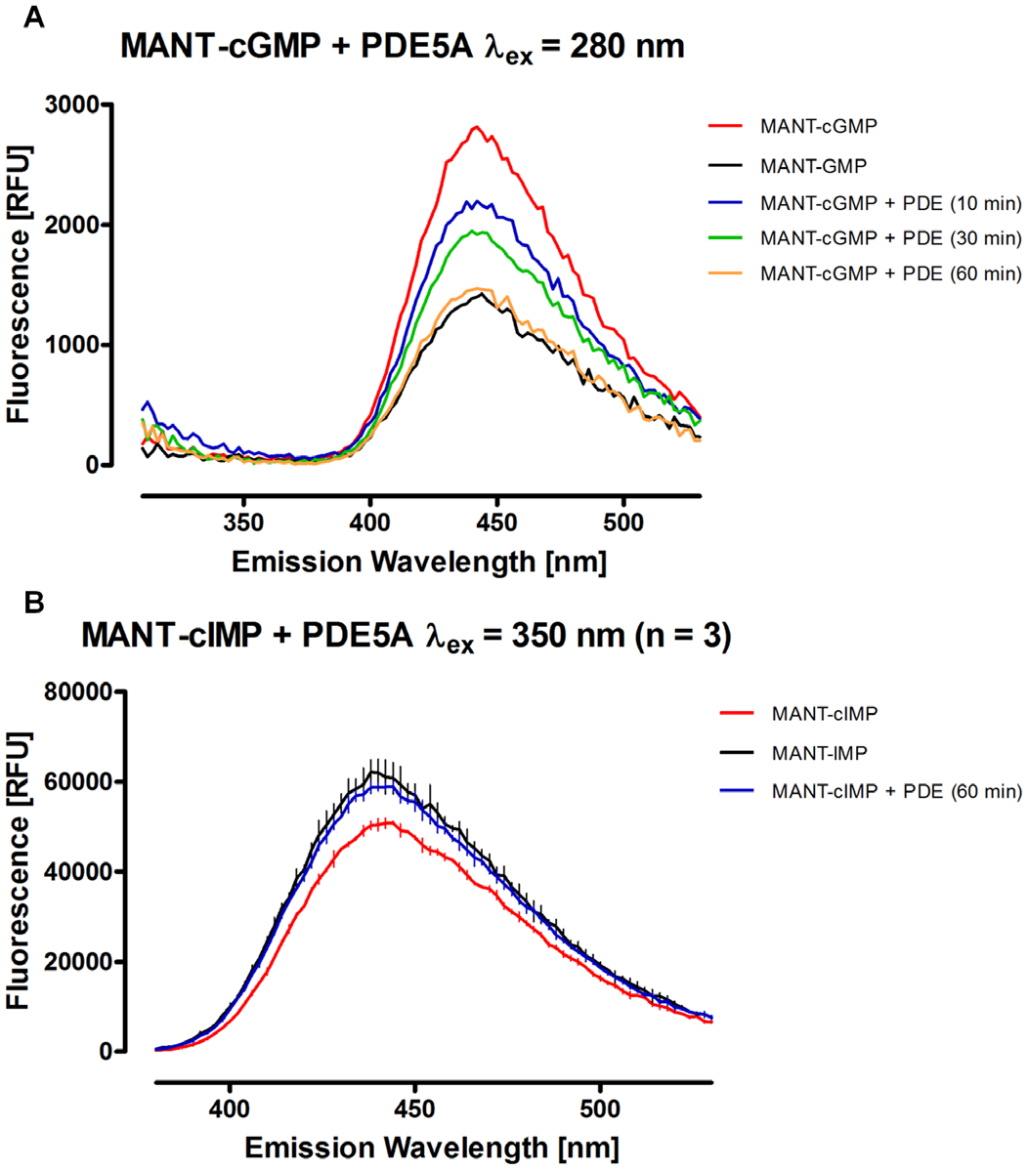
Emission spectra of MANT-cGMP (A) and MANT-cIMP (B) incubated with PDE5A compared to the respective substrate and product standards. Incubation times were as stated within the particular graph. Concentrations of enzyme and substrates were as specified in the materials and methods section. The background fluorescence including fluorescence caused by the buffer has been subtracted from each graph. Note, that relative fluorescence units (RFU) are not necessarily comparable between the particular figures.

**Figure 3:**
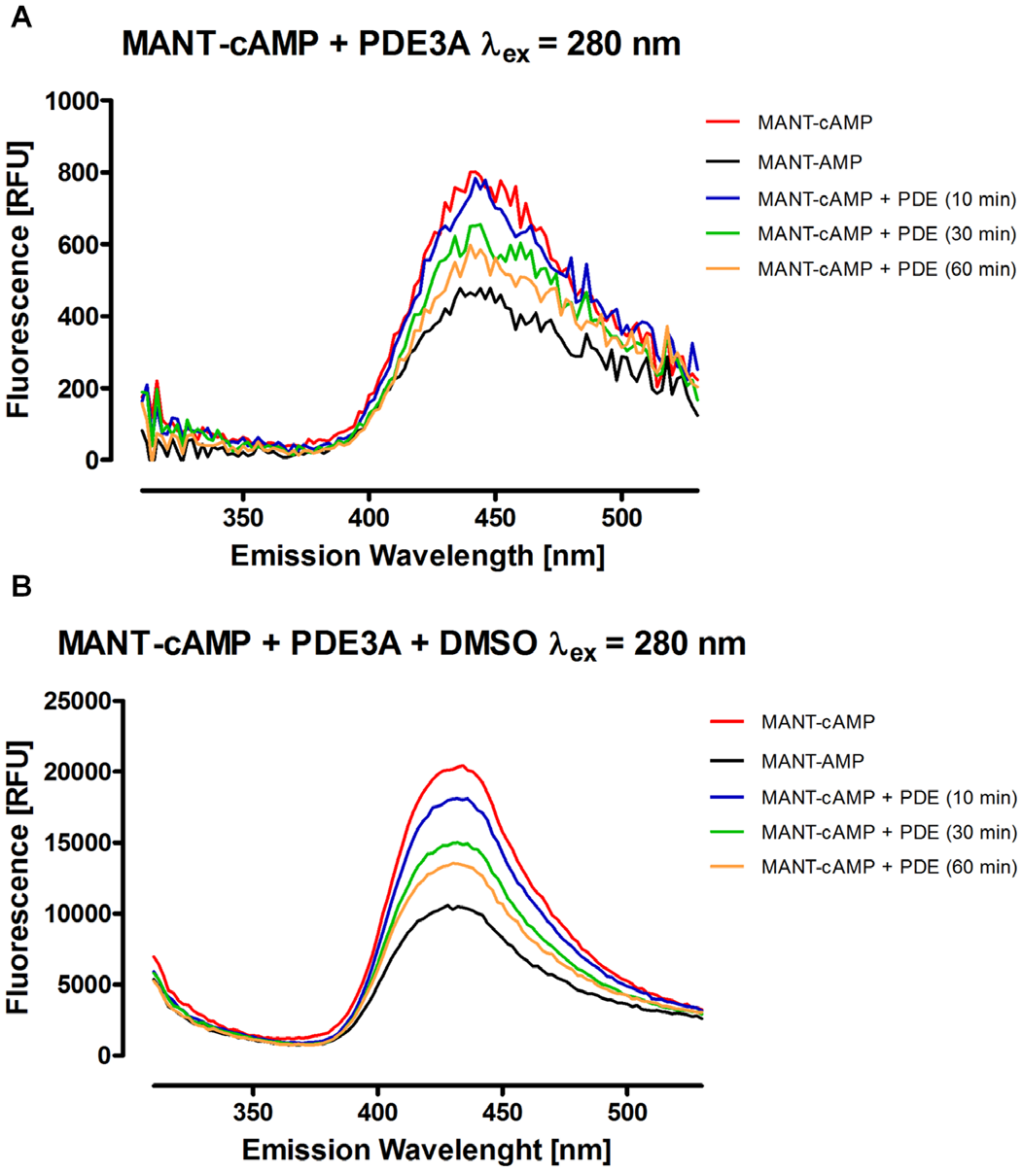
Emission spectra of MANT-cAMP incubated with PDE3A compared to the substrate and product standards. In **A**, the samples were directly measured after stopping the enzyme reaction, whereas in **B**, 200 µl of DMSO were added to each sample before performing the fluorescence measurement. Incubation times were as shown above. Concentrations of enzyme and substrates as well as the further reaction conditions were as specified in the materials and methods section. The background fluorescence including fluorescence caused by the buffer has been subtracted from each graph. Note, that relative fluorescence units (RFU) are not necessarily comparable between the particular figures.

**Figure 4:**
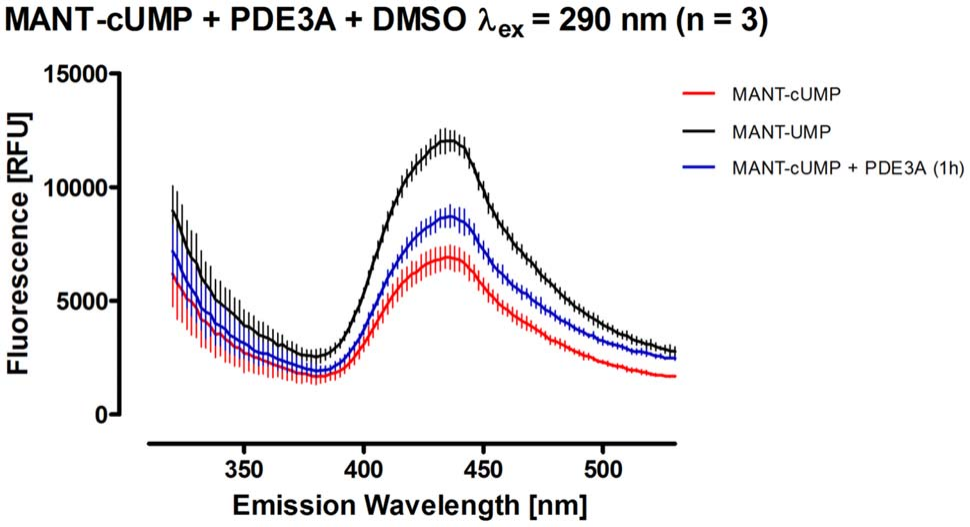
Emission spectrum of MANT-cUMP incubated with PDE3A compared to the substrate and product standards. Incubation time was one hour. Concentrations of enzyme and substrates were as specified in the materials and methods section. The background fluorescence including fluorescence caused by the buffer has been subtracted from each graph. Note, that this figure’s relative fluorescence units (RFU) are not necessarily comparable to the other figures.

Using the parameters specified above for fluorescence detection, the end point assay described in the methods section was performed for each combination of MANT-cNMP and PDE available to us. Emission spectra were detected to analyze the respective PDE activity. Figure 2 shows the results of the reaction of PDE5A with MANT-cGMP (a) and MANT-cIMP (b). Whereas fluorescence was decreased during degradation of MANT-cGMP, MANT-IMP showed a higher fluorescence than MANT-cIMP as anticipated from the previous experiments. MANT-cGMP was hydrolyzed by PDE5A until a nearly complete conversion was accomplished after 60 minutes. MANT-cIMP was completely degraded within 60 minutes as well. When performing the experiment with MANT-cAMP and PDE3A for reaction times of 10 to 60 minutes, an incomplete hydrolysis was observed (figure 3). Most notably, comparing figure 3a to 3b clearly emphasizes the advantage of DMSO as a fluorescence amplifier for MANT-cAMP/MANT-AMP, as DMSO did not only lead to a much smoother curve but also improved the methods’ discriminatory power. Similar to MANT-cIMP, MANT-cUMP cleavage by PDEs produced increased fluorescence as shown for PDE3A (figure 4).

The emission spectra from each combination of PDE and MANT-cNMP were used to calculate respective turnover rates. For this purpose, the emission of each sample after 60 minutes of incubation was compared with the respective standards. The substrate standard of 10 µM MANT-cNMP and the product standard of 10 µM MANT-NMP were defined as 0% turnover and 100%, respectively. The PDEs themselves did not have any impact on the endogenous fluorescence of MANT-substituted nucleotides, which is imperative for this approach (data not shown). Moreover, due to the high specific activity and purity of the enzymes used, the total amount of protein in the samples could be kept rather small. The data acquired by this calculation are compared with data from the literature [29], obtained by high performance liquid chromatography/mass spectrometry-analytics using native cNMPs (table 2). Reaction conditions, buffers, and the concentrations of the cofactors of PDE1, Ca^2+^ and calmodulin, used in the present assay with MANT-cNMPs were identical to those used with cNMPs [29] to ensure direct comparability. Turnover was calculated as mean reaction rate in nmol/mg/min determined after a reaction time of 60 minutes for better comparison to previous data on native cNMPs [29], the values are, therefore, not necessarily comparable with initial reaction rates stated elsewhere [25], [26] and do not exactly reflect *in vivo* hydrolysis. All turnover rates were validated semi-quantitatively by means of TLC.

**Table 2:** Comparison of the activity of various PDEs on MANT-cNMPs as assessed by the fluorescence assay described here and on native cNMPs [29] assayed by mass spectrometry.

		cAMP or MANT-cAMP	cGMP or MANT-cGMP	cCMP or MANT-cCMP	cUMP or MANT-cUMP	cIMP or MANT-cIMP
PDE1B	Fluorescence assay	**n.d.**	**311±17.5**	n.d.	n.d.	**286±231**
PDE1B	Mass spectrometry [29]	226	326	n.d.	9.60	310
PDE3A	Fluorescence assay	**196±15.1**	60.5±17.5	n.d.	**81.7±16.3**	**267±78.3**
PDE3A	Mass spectrometry [29]	121	20.3	n.d.	102	121
PDE5A	Fluorescence assay	n.d.	374±55.9	n.d.	n.d.	**290±94.9**
PDE5A	Mass spectrometry [29]	219	403	n.d.	18.7	415
PDE9A	Fluorescence assay	33.7±48.6	**148±16**	n.d.	n.d.	14.8±30.2
PDE9A	Mass spectrometry [29]	30.5	13.7	n.d.	36.6	26.7

Values are given as mean reaction rate detected over 60 minutes in [nmol/mg/min] (mean ± SD). n = 3, n.d. = not detected. Spectra used for calculation of the values printed bold are shown in figure S5.

We found similar turnover rates for native and MANT-substituted cNMPs for most PDEs with some remarkable exceptions. PDE1B hydrolyzed cAMP very well, but did not hydrolyze MANT-cAMP at all. Whereas the turnover of MANT-cGMP and cGMP as well as MANT-cIMP and IMP were similar, the low cUMP hydrolysis could not be confirmed with MANT-cUMP. PDE3A was able to hydrolyze any of the tested MANT-cNMPs except MANT-cCMP with reaction rates being slightly different between native and MANT-labeled nucleotides. PDE5A showed similar behavior as PDE1B, not being able to cleave MANT-cAMP and MANT-cUMP while a turnover for the respective native nucleotides is specified in literature [29]. PDE9A cleaved cUMP, but did not cleave MANT-cUMP and the turnover of MANT-cGMP was significantly higher than stated for native cGMP. Finally, none of the tested PDEs was able to hydrolyze MANT-cCMP.

### Continuous Fluorescence Detection Assay and Calculation of Kinetic Parameters

**Figure 5:**
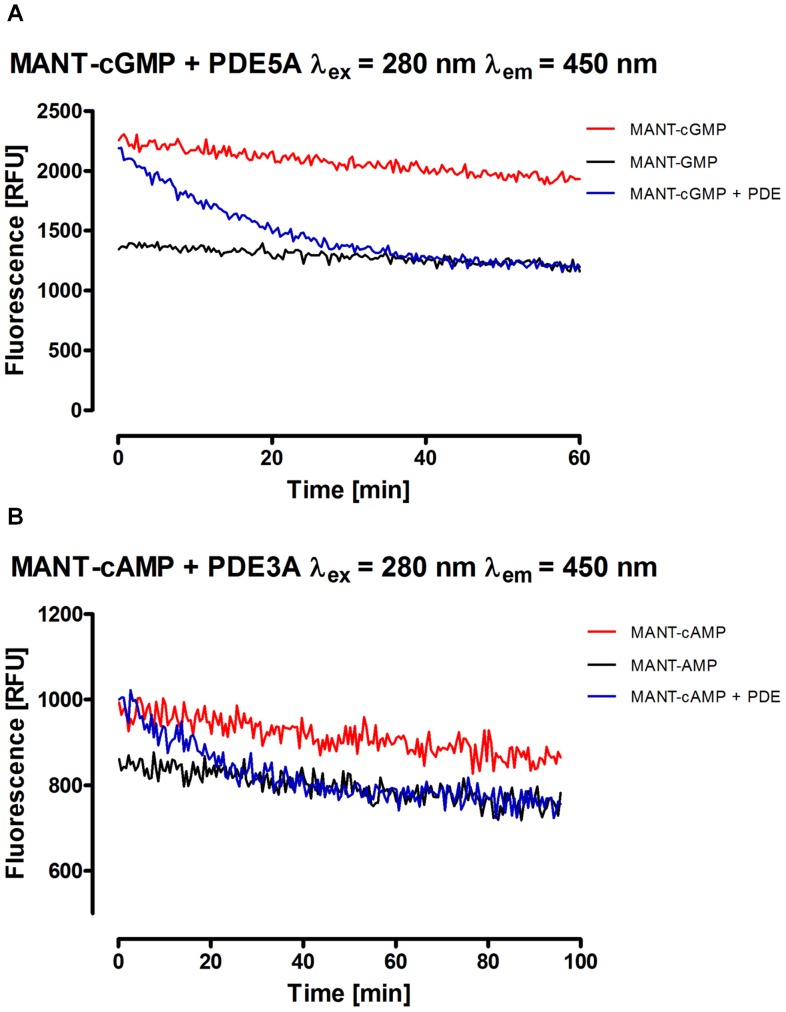
Kinetic analysis of the degradation of MANT-cGMP (A) and MANT-cAMP (B) by PDE5A/PDE3A *via* continuous fluorescence monitoring. After starting the reaction process by adding the respective enzyme to the samples, fluorescence has been determined every 20 sec (A) or 30 sec (B). Excitation was performed at a wavelength of 280 nm, emission was detected at 450 nm. Further reaction conditions were as described in the materials and methods section. Note, that relative fluorescence units (RFU) are not necessarily comparable between the particular figures.

In addition to the end point assay, a continuous fluorescence detection assay was developed to characterize the reaction course more closely and to calculate kinetic parameters for MANT-cNMP hydrolysis. Thus, fluorescence detection was performed during the reaction and fluorescence was plotted against time. Figure 5a shows the reaction course of MANT-cGMP with PDE5A. Complete hydrolysis was achieved within 60 minutes, which is consistent to the findings of the end point assay. As shown in Figure 3, MANT-cAMP and MANT-AMP differ only slightly in fluorescence in the absence of DMSO. However, DMSO effectively inhibited the hydrolysis of MANT-cNMPs by PDEs (data not shown). Hence, adding DMSO before starting the reaction in this continuous fluorescence detection experiment was not feasible. As a consequence, this assay had a much lower sensitivity when using MANT-cAMP as substrate (figure 5b). The slight loss of fluorescence of the MANT-cNMP and MANT-NMP standards during the course of the experiment could be attributed to evaporation and was not caused by bleaching (data not shown). Possibly, quenching effects due to increasing concentrations of the MANT nucleotides play a role.

**Figure 6:**
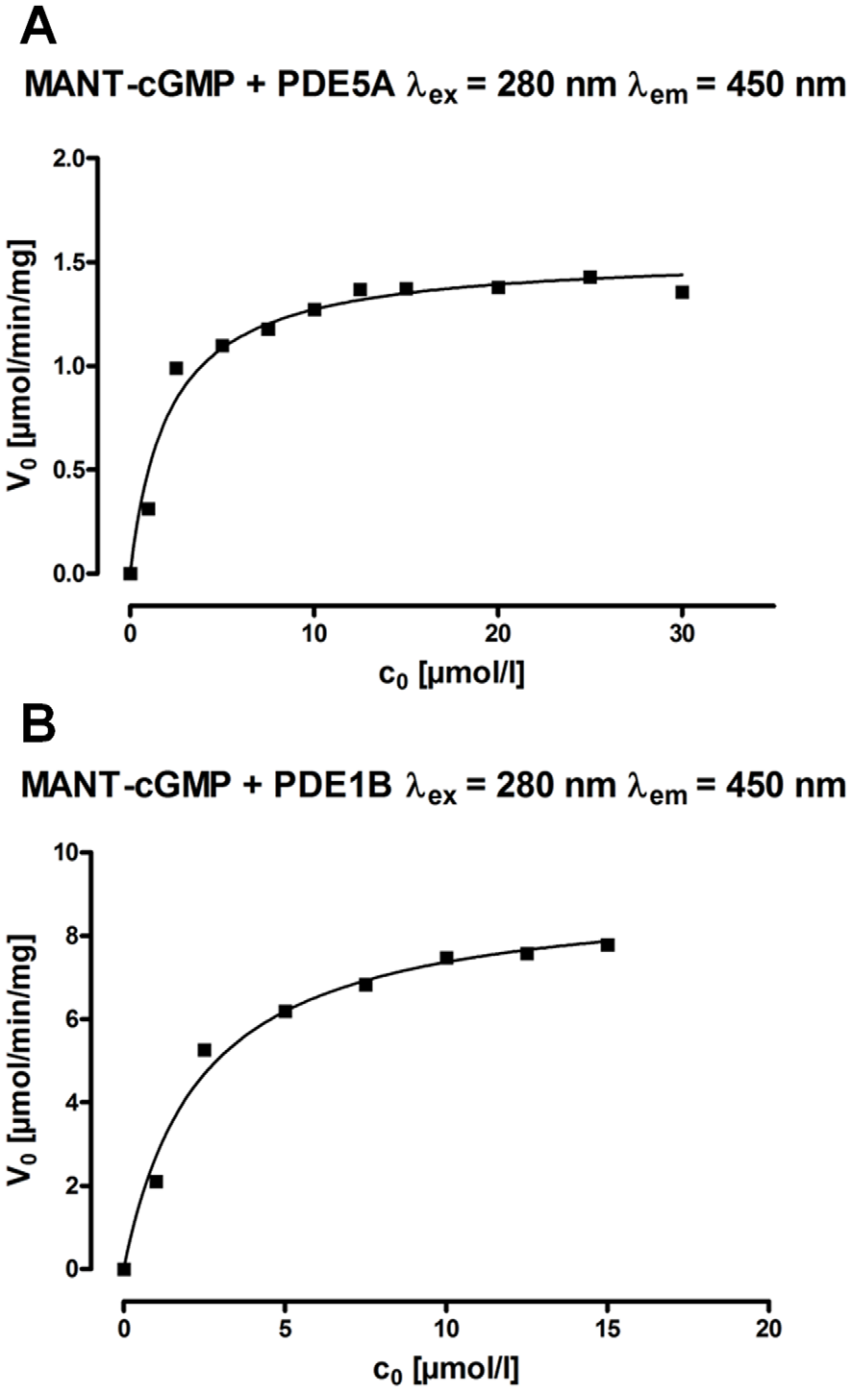
Michaelis Menten kinetics of the hydrolysis of MANT-cGMP by PDE5A (A) and PDE1B (B) determined by continuous fluorescence monitoring. The v_0_/c_0_-diagrams displayed here led to the K_m_ and V_max_ values shown in table 3.

Compared to other assays, the continuous fluorescence method allows to calculate the reaction rate at any point of the reaction directly from the graphs as shown in figure 5 by differentiation. Here, the calculation was exemplarily performed for PDE1B and PDE5A with MANT-cGMP. For both enzymes and various starting concentrations of MANT-cGMP from 1–30 µM (PDE5A) or 1–15 µM (PDE1B) fluorescence/time-diagrams were generated (not shown, analogous to figure 5A). After converting the fluorescence values to substrate concentrations on the basis of the substrate and product standard curves, c/t-diagrams were generated. By differentiating each of these c/t-diagrams with respect to t, the respective initial reaction rate (v_0_) values were calculated. Thus, the v_0_/c_0_-diagrams displayed in figure 6 were elaborated, leading to the K_m_ and V_max_ values shown in table 3, where they are compared to values for native cGMP from literature [25]. Regarding to both enzymes, K_m_ and V_max_ values for cGMP were not substantially different from the values for MANT-cGMP assessed here.

**Table 3:** K_m_ and V_max_ values of PDE1B and PDE5A for MANT-cGMP calculated from the v0/c0-diagrams displayed in figure 6 compared to values for cGMP from literature [25].

	PDE5A		PDE1B	
	MANT-cGMP	cGMP [25]	MANT-cGMP	cGMP [25]
K_m_ [µmol]	2.1	2.9–6.2	2.4	1.2–5.9
V_max_ [µmol/min/mg]	1.6	1.3	9.1	30

### Analysis of Competitive Inhibition of PDE5A Using MANT-cGMP and Native cNMPs

**Figure 7:**
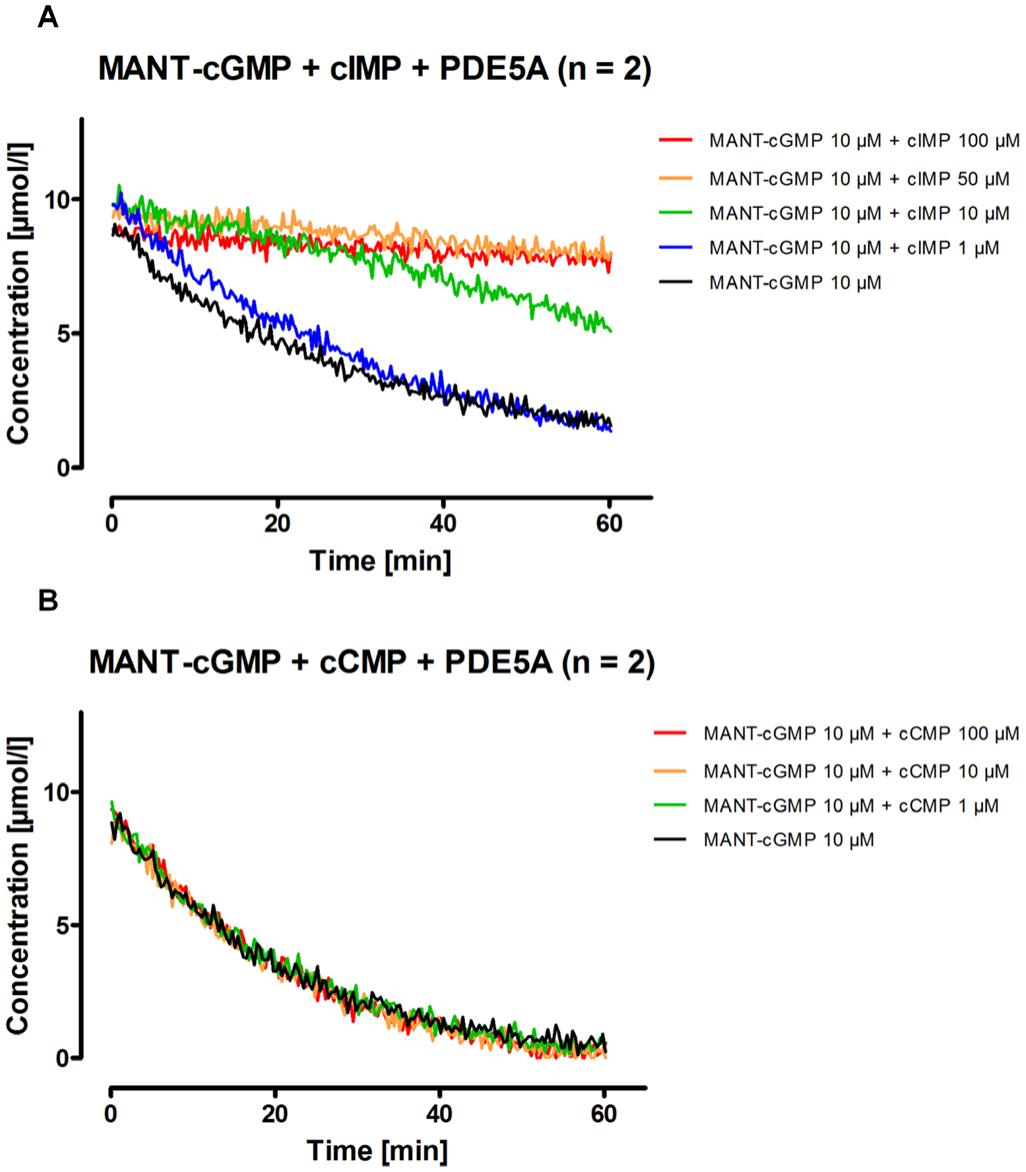
Inhibition of PDE5A mediated hydrolysis of MANT-cGMP by cIMP (A) and cCMP (B). Different concentrations of cIMP or cCMP were added to the reaction batches before starting the reaction (final concentrations given above). Fluorescence was monitored continuously. Excitation was performed at 280 nm, emission was detected at 450 nm. The resulting fluorescence values have been converted to concentrations of remaining MANT-cGMP using calibration graphs. Further reaction conditions were as described in the materials and methods section.

Various native cNMPs were titrated with MANT-cGMP and then incubated with PDE5A to investigate whether MANT-cNMPs are useful tools for inhibitor studies and whether they compete with native cNMPs as substrates for hydrolysis. A continuous fluorescence detection as specified above was then performed and the detected concentration of remaining MANT-cGMP was plotted against reaction time. No native cNMP influenced the fluorescence of any of the tested MANT-cNMP under the given conditions (data not shown), thus allowing to determine the MANT-cGMP turnover. Results are shown in figure 7 regarding the titration of cIMP and cCMP. Being a good substrate of PDE5A [29], cIMP also inhibited PDE5A effectively in hydrolyzing MANT-cGMP (figure 7a). In contrast, cCMP (figure 7b), cAMP and cUMP (data not shown) titrated up to concentrations of 100 µM, which was 10-fold the concentration of the substrate MANT-cGMP, did not inhibit PDE5A.

### Thin Layer Chromatography

To validate and visualize the results that were acquired *via* the end point assay for PDE activity analysis, samples were analyzed by TLC after the direct fluorescence detection was completed. In figure S4, the experiment using MANT-cAMP and PDE3A is shown exemplary. Samples were visualized at 366 nm and their respective R_f_-values were determined. Then, each spot was scraped off the TLC plate and eluted. After adding DMSO, a further emission spectrum was recorded. The resulting emission spectra are shown in figure S4b for MANT-cAMP and PDE3A. The R_f_-values determined for each MANT-cNMP and MANT-NMP are shown in table S1 and compared to values reported by Hiratsuka [40] who used a similar method to analyze purity of MANT nucleotides. The results of the TLC-experiments were congruent with the direct fluorescence detection for MANT-cAMP, MANT-cGMP, MANT-cUMP and MANT-cIMP. In contrast, MANT-cCMP was hydrolysis-resistant up to 24 hours.

## Discussion

In this study, we describe the synthesis of new fluorescent analogs of cIMP as well as cUMP by introducing MANT as a fluorescent probe at the 2′-O-ribosyl position. MANT-IMP, MANT-CMP, and MANT-UMP were synthesized as product standards and calibrators for the development of our improved fluorescence-based PDE assays. Besides this, MANT-cAMP, MANT-cGMP, and MANT-cCMP were obtained in highest purities of >99% (by HPLC). The analyzed MANT-cNMPs exhibit characteristics that are typical for MANT-nucleotides, such as emission maxima at 440–450 nm, high quantum yield, high resistance to photobleaching and distinct blueshift in the presence of organic solvents [39], [40], [41]. However, the particular heat stability described for MANT-c-diGMP [39] could not be confirmed by us, here. In our hands, 2′-O-ribosyl esters (like acetyl-, butyryl-, succinyl-, benzoyl-, or MANT-substituents) of cNMPs are prone to hydrolysis by esterases, pH >7 and heat (data not shown). As ideal excitation wavelengths are given inconsistently in the literature with respect to MANT-cAMP and MANT-cGMP [40], [41], we specified conditions for fluorescence detection of each MANT-cNMP, leading to emission characteristics significantly different from the respective MANT-NMP. Furthermore, instead of dimethylformamide [39], [40], we show that the less toxic substance DMSO is also appropriate for this purpose.

Taking advantage of the difference in fluorescence between MANT-cNMPs and MANT-NMPs, we developed two improved methods for the analysis of the substrate-specificity of PDEs. Studying purified human recombinant PDEs 1B, 3A, 5A and 9A, new findings on their substrate specificity regarding MANT-nucleotides were obtained. In contrast to MANT-substituted nucleoside 5′-triphosphates, which are not accepted as substrates by various bacterial and mammalian nucleotidyl cyclases and are, in fact, inhibitors [32], [33], [34], [35], [37], certain MANT-cNMPs are good substrates for different human PDEs [29]. This is particularly true for PDE5A and PDE1B whose kinetic parameters regarding MANT-cGMP are close to the values for native cGMP [25]. However, there are notable exceptions regarding MANT-cAMP and MANT-cUMP, which did not act as substrates of PDE1B and 5A whereas the native nucleotides are effectively hydrolyzed [29]. In case of MANT-cUMP this may be attributed to limitations in sensitivity, as native cUMP is not hydrolyzed at high rates by these enzymes. The fact that PDE3A was the only tested PDE that hydrolyzed MANT-cUMP besides native cUMP is nonetheless a hint that cUMP may be a physiological substrate of this particular PDE. As PDE3A is located in cardiac myocytes [25], it may be identical with the forgotten cUMP-hydrolyzing activity described by Hardman and Sutherland almost 50 years ago [28]. In case of MANT-cAMP, the MANT fluorescent probe may mediate a certain steric inhibition of some PDEs and a remaining, low MANT-cAMP turnover may then be below the detection limit of the fluorescence assay. This view is compatible with data published by Hiratsuka [40], who detected a hydrolysis rate reduction to about 15% in comparison to native cAMP when using ANT- or MANT-cAMP as substrate of a bovine PDE. It is remarkable that none of the tested PDEs is capable of hydrolyzing MANT-cCMP. This finding is consistent with literature on native cCMP [29] and may point to the existence of other elimination mechanisms for cCMP. As multidrug resistance proteins 4 and 5 are known to transport cAMP and cGMP effectively [47], [48], [49], export from the cell by organic anion transporters or multidrug resistance proteins may play a role for cCMP elimination as well.

The fluorescence of any studied MANT-cNMP did not interfere with any native cNMP tested here, rendering MANT-cNMPs excellent tools not only to study ortho- or allosteric inhibition of PDEs but also to examine regulatory domains as well as crosstalk and competition between the particular nucleotides. Regarding PDE2, a stimulatory, cGMP-binding GAF domain is described that plays an indispensable role in the regulation of this enzyme [25]. As crosstalk between cAMP and cGMP is well established [25], [26], [27], this may be also of interest regarding the emerging second messengers cCMP and cUMP.

The assay introduced here offers various advantages compared to the HPLC-MS method described in the literature [29]. It does not require highly sensitive mass spectrometry equipment as detection can be performed with a standard fluorimeter. Furthermore, large numbers of samples can be analyzed within a substantially shorter period of time. Moreover, the continuous fluorescence monitoring method allows evaluation of the reaction rate at any given time during the course of the reaction, which renders it also advantageous in comparison to radiometric assays. This is especially helpful to perform inhibitor studies or carry out regulatory experiments, as time-delayed titration of putative inhibitors or regulatory molecules is possible and their effects can be directly observed. Finally, the results obtained via direct fluorescence detection may be validated by TLC.

We also need to clearly emphasize the limitations of our methods described here. In addition to the fact that some MANT-cNMPs do not behave exactly like the respective native cNMPs in terms of substrate specificity of several PDEs, there are also relevant limitations in terms of sensitivity. The lower detection limit is reached at substrate concentrations of about 1 µM in reference to MANT-GMP using the equipment available to us. When lower concentrations were used, distinction between MANT-cGMP and MANT-GMP was not reliable anymore. Concerning MANT-cAMP and MANT-AMP with an endogenously smaller difference in fluorescence, sensitivity is even lower. Although sensitivity can be markedly improved by addition of DMSO, this is only feasible for the endpoint assay, but not for continuous fluorescence monitoring and not for all MANT-cNMPs. This limitation of sensitivity, especially when nucleotides that provide lesser discriminatory power such as MANT-cIMP are used, causes the deviations of the reaction rates being spread rather widely (table 2). As some human PDEs possess K_m_ values below substrate concentrations of 1 µM [25], kinetic analysis of such enzymes will be difficult using the methods described here. It must be stated that although the fluorescence assay is suitable to analyze purified recombinant enzymes, application on tissues or cell preparations will be impeded by the low sensitivity compared to our HPLC-MS method and conventional radiometric methods. Nonetheless, as mechanisms of elimination are neither completely understood for cCMP nor for cUMP, the present assay provides a method for the analysis of numerous PDEs with moderate technical requirements.

Our present work has a technical focus, and we have discussed the advantages and disadvantages of the fluorescence method compared to HPLC-MS and, to a very limited extent, radiometric methods. Beyond the technical aspect, our work has also broad biological implications for future studies. It is now clear that future studies on cNMP-degrading PDEs must include substrates beyond the “standard” cNMPs cAMP and cGMP and their corresponding MANT-derivatives. cIMP and cyclic pyrimidine nucleotides may constitute important physiological substrates and/or regulators of PDEs. As a result of our recent study [29] and our present study, a systematic kinetic re-analysis of cNMP-degrading PDEs is required. Evidently, this is a substantial amount of work that cannot be accomplished very rapidly and by a single laboratory, specifically if one keeps in mind that cCMP and cUMP may also constitute hitherto unrecognized positive and/or negative allosteric PDE regulators. Our negative data on allosteric regulation of selected PDEs by cUMP and cCMP does not exclude the possibility that such regulation exists for some PDEs. This re-analysis of PDEs may also result in a refinement of current PDE classification [25]–[27].

Considering the fact that PDEs are differentially expressed in cells and tissues, the substrate-specificity of these enzymes will also provide important information on the potential biological functions of cCMP and cUMP. It will be particularly important to study the biological role of cUMP in the heart, where the cUMP-degrading PDE3A is expressed [25]–[28]. Moreover, a systematic search for the elusive cCMP-degrading PDE [9], [10] is warranted because our negative data on selected PDEs do not exclude the existence of a cCMP-degrading PDE. At the time being, we can only speculate about the potential nature of a cCMP-degrading PDE. Such an enzyme may constitute a known PDE not yet studied by us at the time being. Alternatively, it may constitute a novel PDE. It is also possible that a known PDE exhibits cCMP-PDE activity only in the presence of as yet unknown protein regulators. The search for the putative cCMP-degrading PDE is not trivial because it is furthermore possible that catalytic activity of such a PDE requires the presence of other regulatory cNMPs. Thus, future research on PDEs will become truly pluridimensional and much more complex in terms of combining various cNMPs at various concentrations with each other.

A sensitive and generally applicable fluorescence-based assay would be most useful for such systematic and pluridimensional PDE studies, and our present study constitutes the first step towards this ambitious goal. Chemical modification of the MANT group or the introduction of different fluorophores may yield fluorescent PDE substrates with better properties than the ones presently available. Docking of fluorescent cNMPs into available PDE crystal structures is important in this regard [25]–[27]. We predict that biochemical and medicinal-chemical research on cNMP-degrading PDEs will substantially increase as a result of our recent studies and yield many surprising results.

## Supporting Information

Figure S1 **Representative excitation spectra of MANT-cNMPs and MANT-NMPs.** Concentration of each nucleotide was 10 µM. Emission was detected at 440 nm. **A-E** show the spectra without DMSO whereas in **F-K** 200 µl of DMSO were added to each 100 µl sample before the spectra were recorded. Final DMSO concentration was 67% (v/v). Note that relative fluorescence units (RFU) are not necessarily comparable between the particular figures. Data shown are the means ± SD of 3 experiments. Note that in some instances, SD values are too small to be seen.
(TIF)

Figure S2 **Emission spectra of MANT-cAMP in various concentrations of DMSO.** A solution of MANT-cAMP (10 µM final concentration) was diluted in 100 µl solvent containing different concentrations of DMSO from 5% to 90% (v/v) in water. Higher concentrations of DMSO led to an increase in fluorescence and a shift of the emission maximum to shorter wavelengths. All other tested MANT-cNMPs and MANT-NMPs showed similar behavior (data not shown).
(TIF)

Figure S3 **Representative emission spectra of MANT-cNMPs and MANT-NMPs.** Concentration of each nucleotide was 10 µM. For each MANT-cNMP/MANT-NMP couple the excitation wavelength providing the best discriminatory power (table 1) was selected. **A-E** show the spectra without DMSO whereas in **F-K** 200 µl of DMSO was added to each 100 µl sample before the spectra were detected. Final DMSO concentration was 67% (v/v). Note that relative fluorescence units (RFU) are not necessarily comparable between the particular figures. Data shown are the means ± SD of 3 experiments. Note that in some instances, SD values are too small to be seen.
(TIF)

Figure S4 **Thin layer chromatography validation experiment with MANT-cAMP and PDE3A.** Incubation times of 10 to 60 minutes are shown. 10 µM MANT-cAMP and 10 µM MANT-AMP were used as standards. After visualizing the samples at 366 nm (**A**), they were scraped off the chromatography plate, eluted and DMSO was added. **B** shows the resulting emission spectra for the respective reaction times. A nonfluorescing spot was used as control. The Rf values for each MANT-cNMP and MANT-NMP tested are displayed in table S1. In **A**, only the blue color channel of the original image is shown and the contrast of the entire image was enhanced via post processing.
(TIF)

Figure S5 **Representative emission spectra of selected MANT-cNMPs and PDEs used for calculation of the reaction rates displayed in** **table 2** **.** Incubation time was 60 minutes, excitation wavelengths were varied to provide ideal discriminatory power for each MANT-cNMP/MANT-NMP couple as stated in table 1. DMSO was added to samples containing MANT-cAMP or MANT-cUMP before recording the emission spectra. Concentrations and further reaction conditions were as described in the materials and methods section. Note, that relative fluorescence units (RFU) are not necessarily comparable between the particular graphs.
(TIF)

Table S1 R_f_-values determined by thin layer chromatography (figure S4) compared to values from literature [40]. (n = 5–8).
(DOC)
