# Supplementary figures and images for: Analysis of Substrate Specificity and Kinetics of Cyclic Nucleotide Phosphodiesterases with N’-Methylanthraniloyl-Substituted Purine and Pyrimidine 3′,5′-Cyclic Nucleotides by Fluorescence Spectrometry
Source: PLoS One. 2013 Jan 14;8(1):e54158. doi: 10.1371/journal.pone.0054158 (PMC3544816; doi:10.1371/journal.pone.0054158)

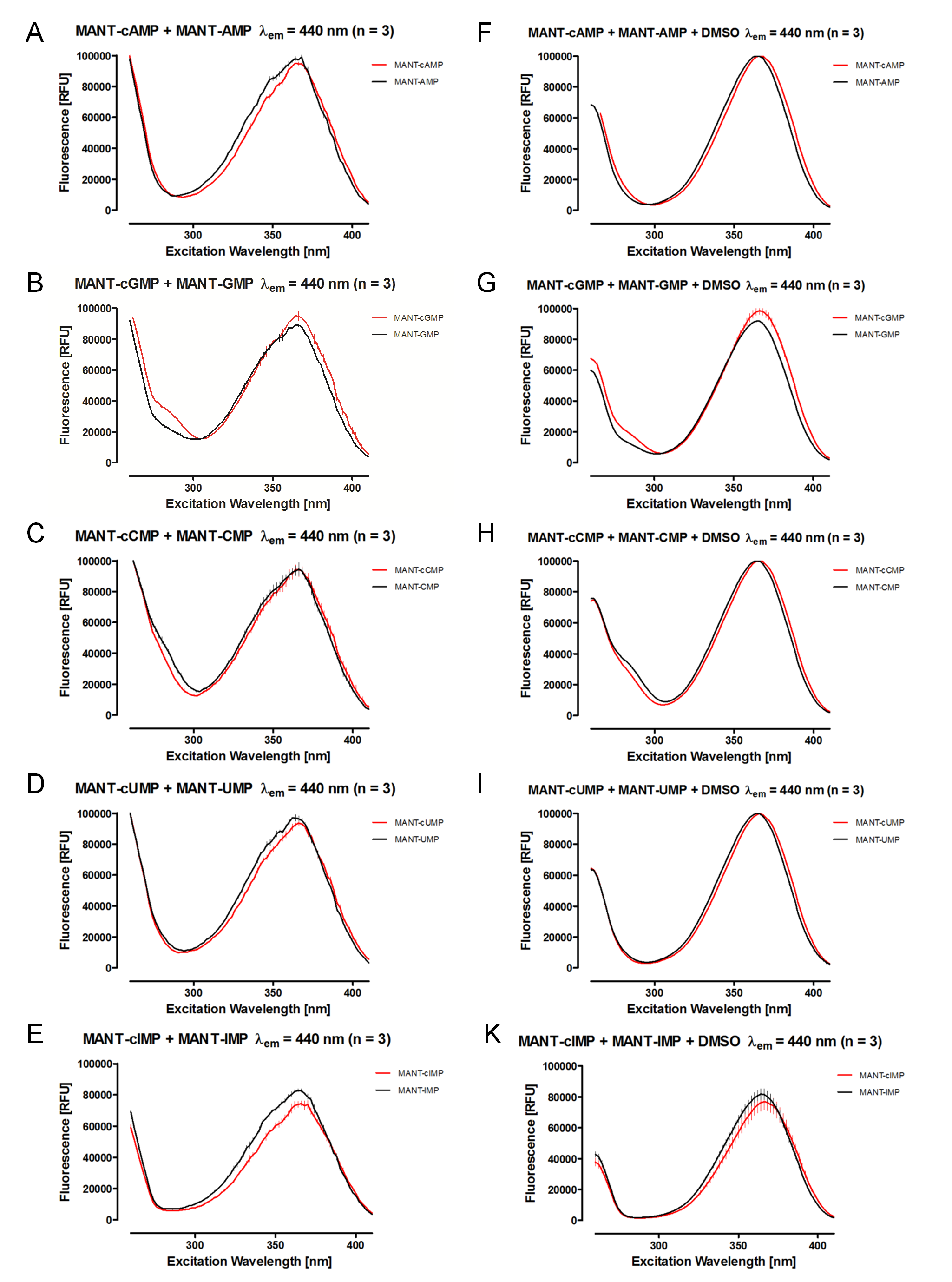

Supplement: Figure S1 — Representative excitation spectra of MANT-cNMPs and MANT-NMPs. Concentration of each nucleotide was 10 µM. Emission was detected at 440 nm. A-E show the spectra without DMSO whereas in F-K 200 µl of DMSO were added to each 100 µl sample before the spectra were recorded. Final DMSO concentration was 67% (v/v). Note that relative fluorescence units (RFU) are not necessarily comparable between the particular figures. Data shown are the means ± SD of 3 experiments. Note that in some instances, SD values are too small to be seen. (TIF) [file pone.0054158.s001.tif]

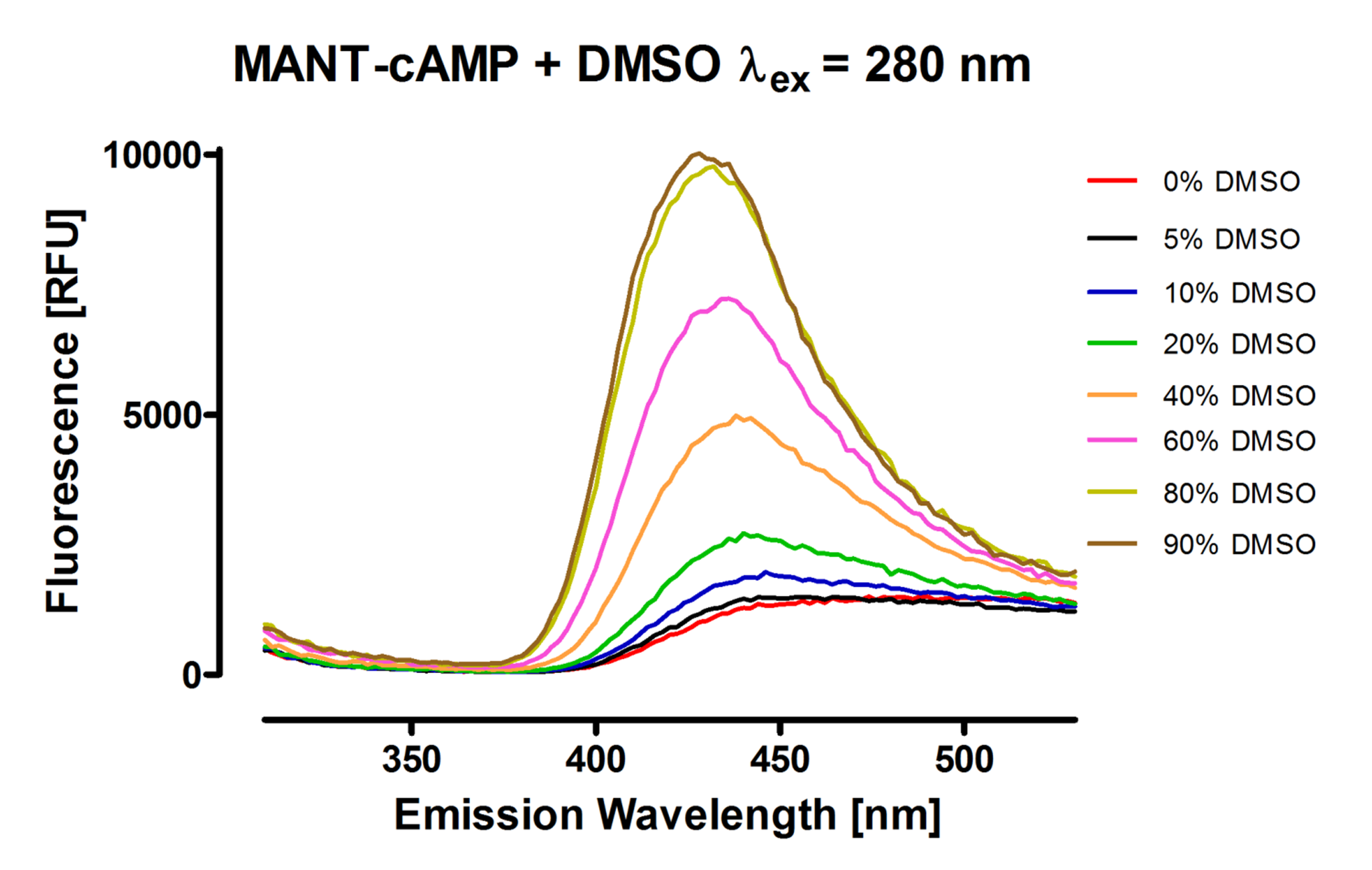

Supplement: Figure S2 — Emission spectra of MANT-cAMP in various concentrations of DMSO. A solution of MANT-cAMP (10 µM final concentration) was diluted in 100 µl solvent containing different concentrations of DMSO from 5% to 90% (v/v) in water. Higher concentrations of DMSO led to an increase in fluorescence and a shift of the emission maximum to shorter wavelengths. All other tested MANT-cNMPs and MANT-NMPs showed similar behavior (data not shown). (TIF) [file pone.0054158.s002.tif]

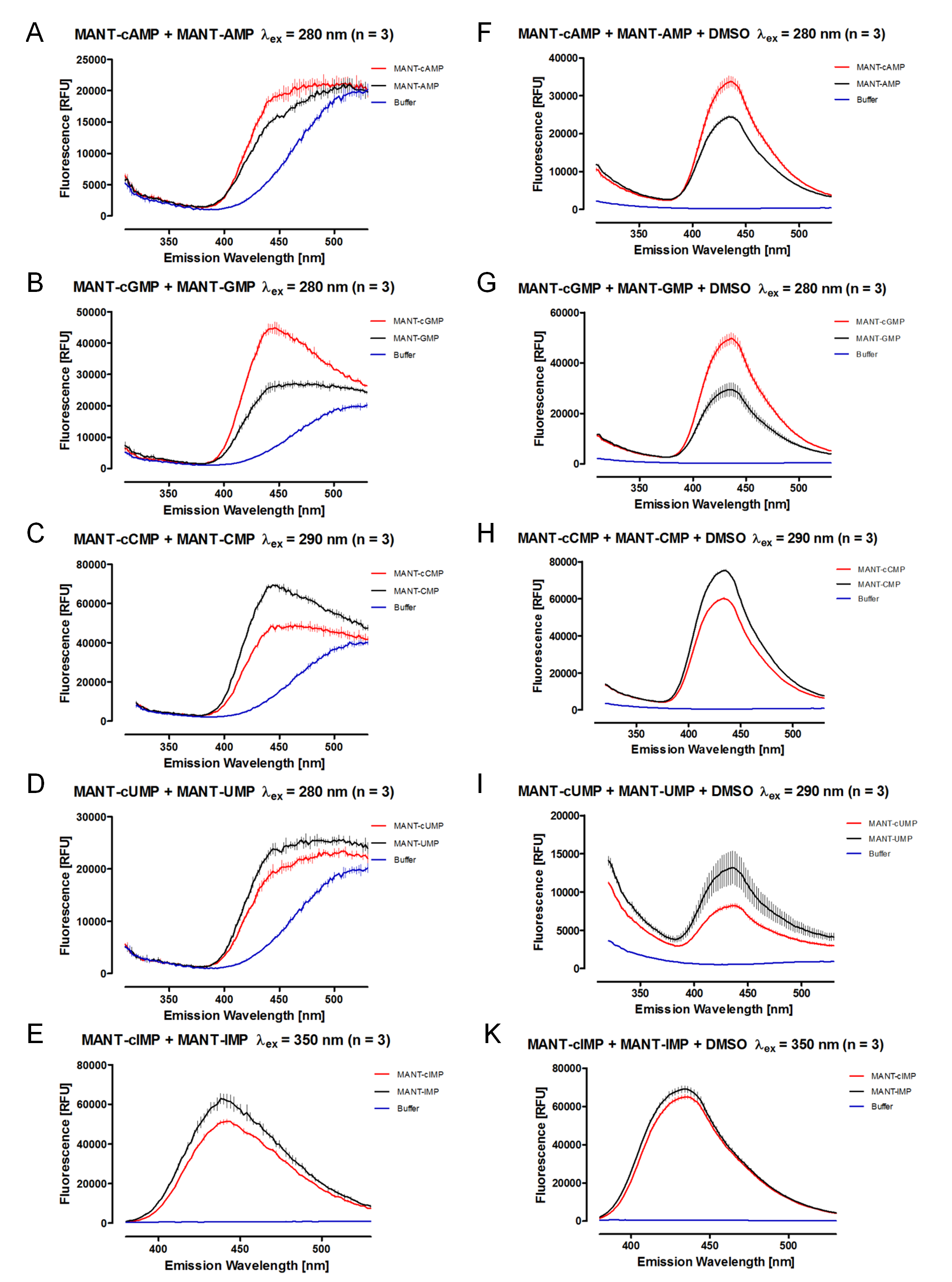

Supplement: Figure S3 — Representative emission spectra of MANT-cNMPs and MANT-NMPs. Concentration of each nucleotide was 10 µM. For each MANT-cNMP/MANT-NMP couple the excitation wavelength providing the best discriminatory power (table 1) was selected. A-E show the spectra without DMSO whereas in F-K 200 µl of DMSO was added to each 100 µl sample before the spectra were detected. Final DMSO concentration was 67% (v/v). Note that relative fluorescence units (RFU) are not necessarily comparable between the particular figures. Data shown are the means ± SD of 3 experiments. Note that in some instances, SD values are too small to be seen. (TIF) [file pone.0054158.s003.tif]

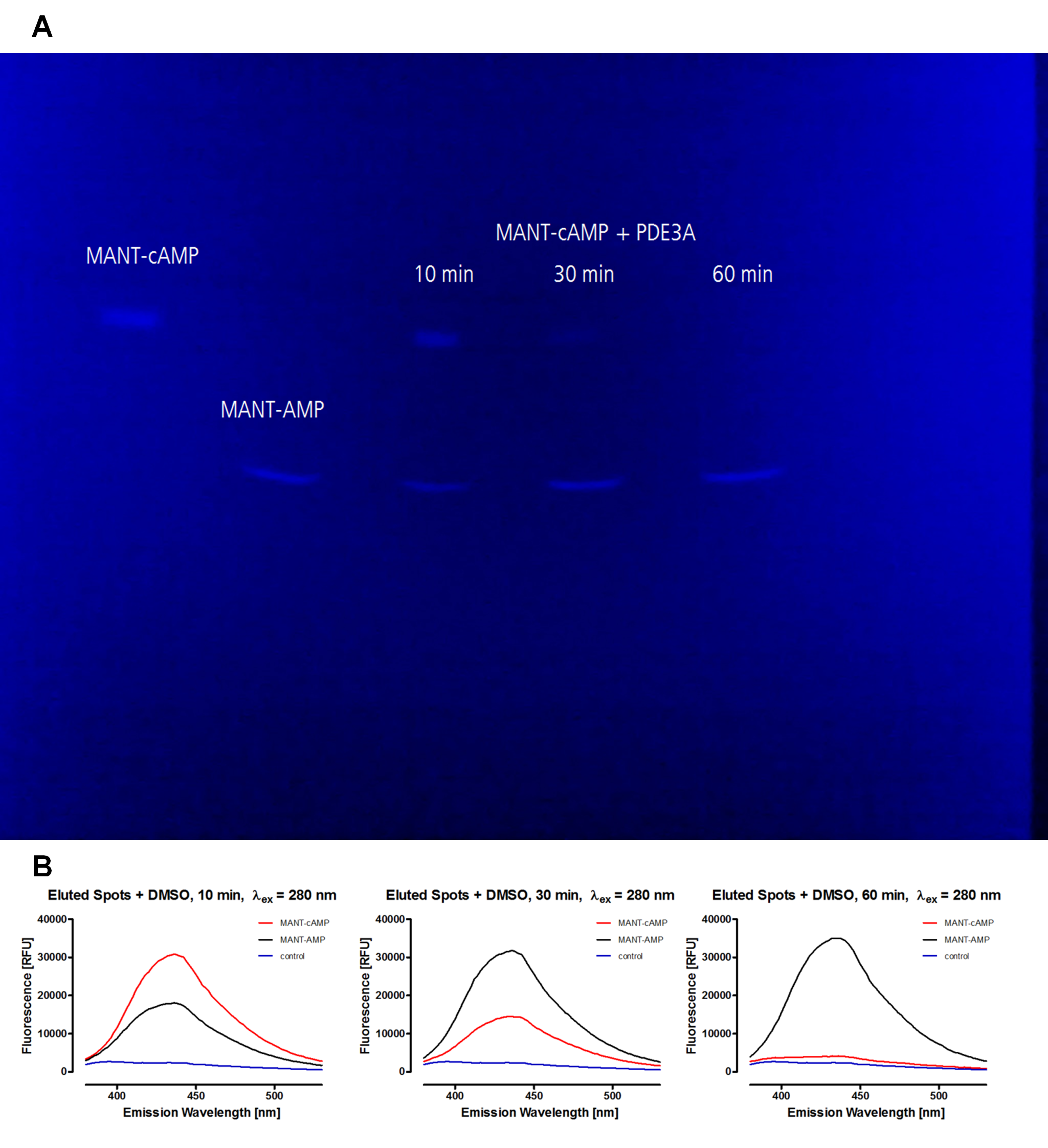

Supplement: Figure S4 — Thin layer chromatography validation experiment with MANT-cAMP and PDE3A. Incubation times of 10 to 60 minutes are shown. 10 µM MANT-cAMP and 10 µM MANT-AMP were used as standards. After visualizing the samples at 366 nm (A), they were scraped off the chromatography plate, eluted and DMSO was added. B shows the resulting emission spectra for the respective reaction times. A nonfluorescing spot was used as control. The Rf values for each MANT-cNMP and MANT-NMP tested are displayed in table S1. In A, only the blue color channel of the original image is shown and the contrast of the entire image was enhanced via post processing. (TIF) [file pone.0054158.s004.tif]

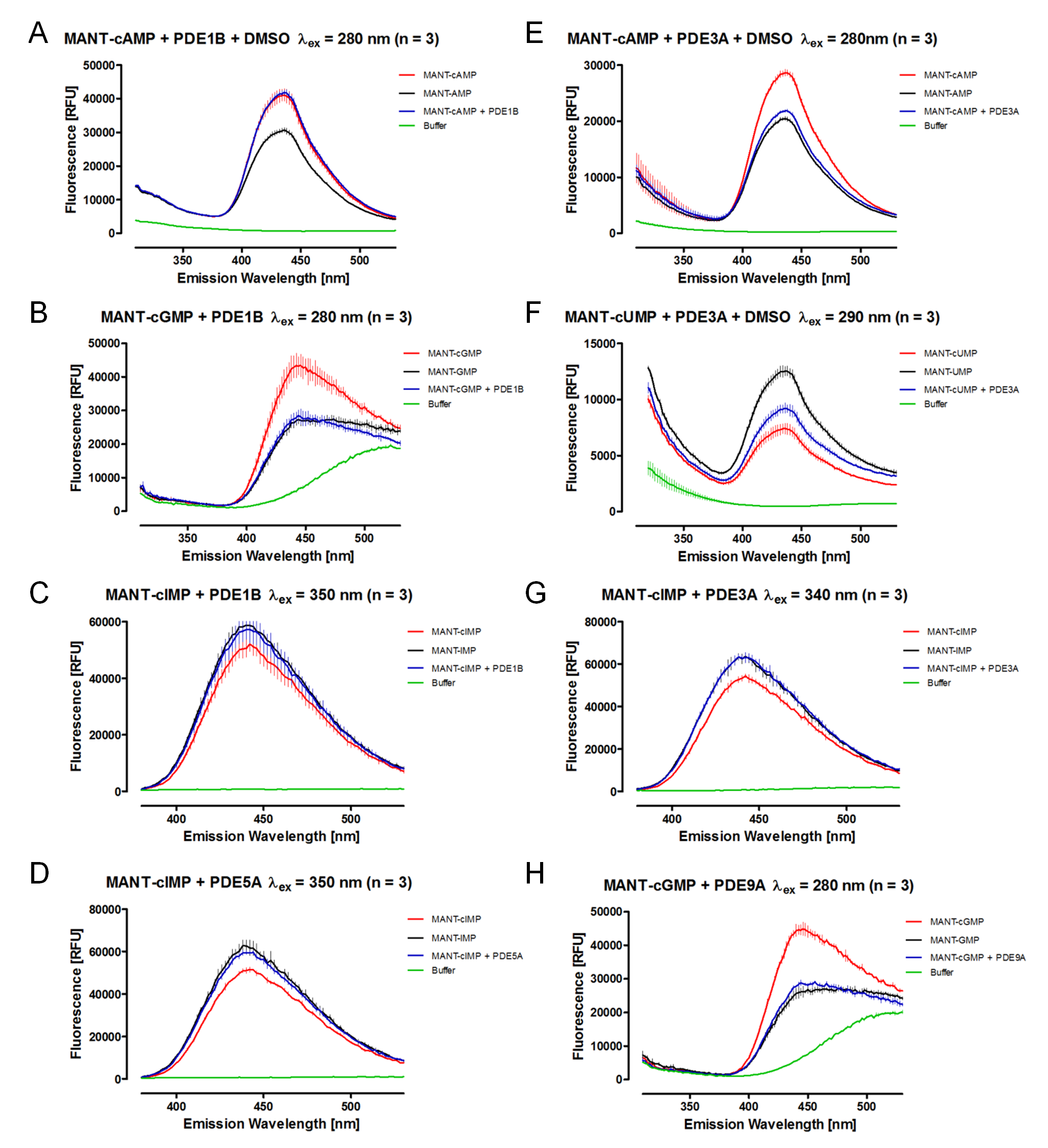

Supplement: Figure S5 — Representative emission spectra of selected MANT-cNMPs and PDEs used for calculation of the reaction rates displayed in table 2 . Incubation time was 60 minutes, excitation wavelengths were varied to provide ideal discriminatory power for each MANT-cNMP/MANT-NMP couple as stated in table 1. DMSO was added to samples containing MANT-cAMP or MANT-cUMP before recording the emission spectra. Concentrations and further reaction conditions were as described in the materials and methods section. Note, that relative fluorescence units (RFU) are not necessarily comparable between the particular graphs. (TIF) [file pone.0054158.s005.tif]
