# Supplementary material for: Analysis of Substrate Specificity and Kinetics of Cyclic Nucleotide Phosphodiesterases with N’-Methylanthraniloyl-Substituted Purine and Pyrimidine 3′,5′-Cyclic Nucleotides by Fluorescence Spectrometry
Source: PLoS One. 2013 Jan 14;8(1):e54158. doi: 10.1371/journal.pone.0054158 (PMC3544816; doi:10.1371/journal.pone.0054158)
Supplement: Table S1 — Rf-values determined by thin layer chromatography (figure S4) compared to values from literature [40]. (n = 5–8). (DOC) [file pone.0054158.s006.doc]

|  | MANT-cAMP | MANT-AMP | MANT-cGMP | MANT-GMP | MANT-cCMP | MANT-CMP | MANT-cUMP | MANT-UMP | MANT-cIMP | MANT-IMP |
| --- | --- | --- | --- | --- | --- | --- | --- | --- | --- | --- |
| Present Assay | 0.80 ± 0.04 | 0.50 ± 0.04 | 0.70 ± 0.05 | 0.40 ± 0.04 | 0.76 ± 0.03 | 0.51 ± 0.04 | 0.74 ± 0.03 | 0.43 ± 0.02 | 0.70 ± 0.02 | 0.45 ± 0.02 |
| Literature [40] | 0.75 | 0.55 | 0.66 | 0.44 |  |  |  |  |  |  |

**Supplemental Table S1.** R_f_-values determined by thin layer chromatography (Supplement figure S4) compared to values from literature [40]. (n = 5 - 8)
